# Novel Materials Synthesis by Mechanical Alloying/Milling

**DOI:** 10.3390/ma15196973

**Published:** 2022-10-08

**Authors:** Joan-Josep Suñol, Lluisa Escoda

**Affiliations:** P2, Escola Politècnica Superior, Campus Montilivi s/n, University of Girona, 17003 Girona, Spain

## 1. Introduction and Scope

Mechanical alloying (MA) and mechanical milling (MM) are based on the ball milling technique/procedure. MA is the milling of several precursors in order to obtain new phases and MM is usually defined as the milling of one precursor to modify its microstructure. MA/MM have been applied to produce advanced materials (amorphous, nanocrystalline, extended solid solution, composite, etc.) [1]. The resulting powders can be consolidated to produce bulk samples or mixed to obtain composites.

The keywords of this Special Issue are:

- Advanced materials;
- Functional properties;
- Powder metallurgy;
- New material synthesis;
- Modelling;
- Milling devices;
- Simulation.

Evidently, as only seven articles were accepted for publication, the set of articles does not cover all the topics and families of materials associated with MA/MM. Nevertheless, it is expected that the manuscripts here published are of interest for researchers working in mechanical alloying. 

## 2. Contributions

The manuscripts of this Special Issue can be classified, taking the materials, processing, and functional properties into account. The information on this is provided in Table 1.

One of the main topics of MA/MM is the development of Fe-rich soft magnetic alloys. Carrillo et al. [2] produced two Fe-rich Fe-Co-Nb-B nanocrystalline (crystallite size ~10 nm) alloys with soft–hard magnetic behavior. The thermal stability of the nanocrystalline phase was checked by differential scanning calorimetry. The optimization of the magnetic response (soft) is obtained after the annealing process at 350 °C in the compacted (Co 10 at.%) powders. This study also compares magnetic parameters (the magnetization of saturation, coercivity, etc.) with those obtained in other Fe-rich alloys.

The temperature of Curie is a limiting parameter for the application of magnetic materials. Manchón-Gordon et al. [3] analyzed the influence of milling time on the Curie temperature of a partially amorphous Fe-Zr alloy. A distribution of the Curie temperature has been found. The average of this temperature increases when the Fe content in the amorphous phase increases. The same effect was also found in the soft magnetic behavior.

With regards to Fe-rich alloys, magnetic refrigeration is an environmental friendly alternative to conventional gas refrigeration. Alleg et al. [4] obtained an Fe-Nb-B alloy by MA and analyzed the hyperfine structure as well as the magnetocaloric effect of these powders (a nanocomposite with nanocrystalline Fe(B), Fe(Nb), and Fe_2_B phases). The critical behavior was calculated and the critical exponent values were close to those expected in the mean field model. The refrigeration capacity was calculated to be ~1.45 J/Kg·K and the refrigeration capacity was ~240 J/Kg. The temperature peak of the magnetocaloric effect was also verified in Ref. [3], with values close to room temperature. It is possible that the alloys produced and magnetically characterized in Ref. [2] are candidates to be studied from an application perspective in systems/devices of magnetic refrigeration.

Mechanical alloying can produce biodegradable metals. These materials are used as load-bearing temporary implants [9]. In an article of this Special Issue [5], Fe-Mn-based alloys were produced by mechanical alloying and consolidated. A key factor was the prediction of the compressibility response of these Fe-Mn-(Cu, W, Co) alloys. Thus, several compaction models were tested and the best results were obtained by applying the Heckel linear model and the Shapiro non-linear model. Experimentally, the achieved final density after stress relief ranged between 72 and 78%. 

MA/MM has been also applied to produce alloys with improved mechanical properties and high resistance to the corrosion. In this Special Issue, two manuscripts on titanium-rich alloys analyzed the results of specimens produced by mechanical alloying and spark plasma sintering. The Ti_2_AlN MAX phase was used (with Ti and AlN as precursors) [6]. The achieved relative density ranged between 91 and 97%. In terms of the mechanical response, the higher measured hardness was 4.3 GPa and the lower measured hardness was 4.0 GPa. One of the main problems is the formation of minor secondary phases such as Ti_5_Si_3_, TiN, Ti_4_AlN_3_, and/or Al_2_O_3._

Kalita and coworkers studied the influence of the addition of molybdenum and thallium on the mechanical behavior of Ti-Nb specimens [7]. It has been found that the mechanical properties are not influenced by the addition of Mo or Ta (in the substitution of Nb content). Oxygen and carbon were detected as interstitial elements favoring a strong solid solution strength level. Likewise, a slight improvement in superelastic behavior was found to be associated with small changes in the strain on the martensitic transformation. 

Mechanical milling has been applied to glassy arsenic monoselenide [8]. Re-amorphization (driven by milling) has also been detected. In this manuscript, the authors state that milling provokes a loss of the molecular network. Ab initio studies can help to determine the microstructure scenario. 

## 3. Conclusions and Outlook

Articles in this Special Issue present results on the production and characterization of materials produced by mechanical alloying/milling. Specifically, there are several articles in which iron-rich alloys have been produced. It has been found that these alloys are suitable candidates for the development of soft magnetic materials, for their application as sensors or in magnetic cores, and for use in magnetic cooling devices. Fe-Mn-based alloys, whose biodegradability makes them candidates for use in medical applications, have also been analyzed. Some studies have analyzed bulk specimens obtained by compaction or spark plasma sintering. This last technique has been applied in Ti-based alloys. Another study has analyzed the influence of mechanical milling on the molecular network behavior of glassy arsenic monoselenide. It should be noted that none of the articles were associated with high-entropy alloys, or with the procurement of massive ones via new additive manufacturing techniques.

The editors, however, hope that the set of articles is of interest among researchers producing alloys and compounds by mechanical alloying/milling. Scientific literature analysis confirms that there are multiple recent research works which apply this material development procedure/technique [10,11,12].

## Figures and Tables

**Table 1:** Information about materials, processing, and functional properties within the Special Issue manuscripts. Mechanical alloying—MA, mechanical milling—MM, spark plasma sintering—SPS.

Material	Processing	Functional Prop.	Reference
Fe-Co-based	MA + compaction	Soft magnetic	[2]
Fe-Zr	MA	Soft magnetic + magnetocaloric	[3]
Fe-based	MA	Soft magnetic + magnetocaloric	[4]
Fe-Mn-based	MA + compaction	Biodegradability	[5]
Ti-Al-based	MA + SPS	Mechanical	[6]
Ti-Nb-based	MA + SPS	Mechanical	[7]
AsSe glass	MM	Semiconductor	[8]

## References

[1] Suñol J.J. (2021). Mechanical alloying: Processing and materials. Metals.

[2] Carrillo A., Daza J., Saurina J., Escoda L., Suñol J.J. (2021). Structural, thermal and magnetic analysis of Fe_75_Co_10_Nb_6_B_9_ and Fe_65_Co_20_Nb_6_B_9_ nanostructured alloys. Materials.

[3] Manchón-Gordón A.F., Ipus J.J., Blázquez J.S., Conde C.F., Conde A. (2020). Influence of milling time on the homogeneity and magnetism of a Fe_70_Zr_30_ partially amorphous alloy: Distribution of Curie temperatures. Materials.

[4] Alleg S., Chabi T., Bensebaa N., Saurina J., Escoda L., Hlil E.K., Suñol J.J. (2020). Investigation on the critical behavior, magnetocaloric effect and hyperfine structure in the Fe_72_Nb_6_B_20_ powders. Materials.

[5] Bagha P.S., Khakbiz M., Sheibani S., Hermawan H. (2018). Design and characterization of nano and bimodal structured biodegradable Fe-Mn-Ag alloy with accelerated corrosion rate. J. Alloys Compd..

[6] Ammar H.R., Sivasankaran S., Alaboodi A.S. (2021). Investigation of the microstructure and compressibility of biodegradable Fe-Mn-Cu/W/Cu nanostructured alloy powders synthesized by mechanical alloying. Materials.

[7] Salvo C., Chicardi E., García-Garrido C., Poyato R., Jiménez J.A., Mangalaraja R.V. (2021). Study of the influence of sintering atmosphere and mechanical activation on the synthesis of bulk Ti_2_AlN MAX phase obtained by spark plasma sintering. Materials.

[8] Kalita D., Rogal L., Berent K., Góral A., Dutkiewicz J. (2021). Effect of Mo and Ta on the mechanical and superelastic properties of Ti-Nb alloys prepared by mechanical alloying and spark plasma sintering. Materials.

[9] Shpotyuk Y., Demchenko P., Shpotyuk O., Balitska V., Boussard-Pledel C., Bureau B., Lukacova-Bujñakova Z., Balaz P. (2021). High-energy mechanical milling-driven reamorphization in glassy arsenic monoselenide: On the path of tailoring special molecular-network glasses. Materials.

[10] Campari E.G., Casagrande A. (2022). Microstructural study of CrNiCrCoFeMn high entropy alloy obtained by selective laser melting. Materials.

[11] Khitouni N., Hammami B., Llorca-Isern N., Ben Mbarek W., Suñol J.J., Khitouni M. (2022). Microstructure and magnetic properties of nanocrystalline Fe_60-x_Co_25_Ni_15_Si_x_ alloy elaborated by high-energy mechanical milling. Materials.

[12] Da Silva Teixeira R., Vieira de Oliveira R., Freitas Rodriguez P., Mascarenhas J., Figueiredo Pereira Neves F.C., dos Santos Paula A. (2022). Microwave versus conventional sintering of NiTi alloys processed by mechanical alloying. Materials.

